# Identification of potential molecular mechanisms and small molecule drugs in myocardial ischemia/reperfusion injury

**DOI:** 10.1590/1414-431X20209717

**Published:** 2020-07-17

**Authors:** Tao Jiang, Yingcun Liu, Biao Chen, Liangyi Si

**Affiliations:** 1 The Third Clinical Medical College, Chongqing Medical University, Chongqing, China; 2 Department of Cardiovascular Medicine, The Third Affiliated Hospital of Chongqing Medical University, Chongqing, China

**Keywords:** Myocardial ischemia/reperfusion injury, Bioinformatics analysis, Differentially expressed genes, Hub genes, Small molecules

## Abstract

Myocardial ischemia/reperfusion (MI/R) injury is a complex phenomenon that causes severe damage to the myocardium. However, the potential molecular mechanisms of MI/R injury have not been fully clarified. We identified potential molecular mechanisms and therapeutic targets in MI/R injury through analysis of Gene Expression Omnibus (GEO) database. Differentially expressed genes (DEGs) were found between MI/R injury and normal samples, and overlapping DEGs were found between GSE61592 and GSE67308. Gene Ontology (GO) and pathway analysis were performed for overlapping DEGs by Database for Annotation, Visualization and Integration Discovery (DAVID). Then, a network of protein-protein interaction (PPI) was constructed through the Search Tool for the Retrieval of Interacting Genes (STRING) database. Potential microRNAs (miRNAs) and therapeutic small molecules were screened out using microRNA.org database and the Comparative Toxicogenomics database (CTD), respectively. Finally, we identified 21 overlapping DEGs related to MI/R injury. These DEGs were significantly enriched in IL-17 signaling pathway, cytosolic DNA-sensing pathway, chemokine signaling, and cytokine-cytokine receptor interaction pathway. According to the degree in the PPI network, CCL2, LCN2, HP, CCL7, HMOX1, CCL4, and S100A8 were found to be hub genes. Furthermore, we identified potential miRNAs (miR-24-3p, miR-26b-5p, miR-2861, miR-217, miR-4251, and miR-124-3p) and therapeutic small molecules like ozone, troglitazone, rosiglitazone, and n-3 polyunsaturated fatty acids for MI/R injury. These results identified hub genes and potential small molecule drugs, which could contribute to the understanding of molecular mechanisms and treatment for MI/R injury.

## Introduction

Coronary heart disease is one of the leading causes of disability and death worldwide. For patients with acute ST-segment elevation myocardial infarction (MI), timely myocardial reperfusion is the most effective way to reduce acute myocardial ischemic injury and limit the size of MI. However, myocardial reperfusion can further induce cardiomyocyte death, a phenomenon called myocardial ischemia/reperfusion (MI/R) injury. A number of studies have identified key factors mediating MI/R injury, such as oxidative stress, intracellular calcium overload, inflammation, and mitochondrial dysfunction. Although various risk factors have been proven to contribute to MI/R injury, the mechanism still remains unclear. The discovery of abnormal signaling pathways and crucial genes will promote the development of targeted therapies in MI/R injury.

Microarray analysis of gene expression by bioinformatics has been widely used to find crucial genes and biological processes in various diseases. In this study, GSE61592 and GSE67308 were obtained from the Gene Expression Omnibus (GEO) database to find differentially expressed genes (DEGs) and overlapping DEGs between MI/R injury and normal myocardium samples. Subsequent bioinformatics analyses were based on the obtained overlapping DEGs. Functional annotation, pathway, protein-protein interaction (PPI) network, and potential miRNAs, as well as small molecules associated with MI/R injury, were analyzed by bioinformatics methods. These results might contribute to the understanding of underlying molecular mechanisms and the finding of potential drugs for MI/R injury.

## Material and Methods

### Datasets and data preprocessing

The GSE61592 and GSE67308 datasets were downloaded from the GEO database (http://www.ncbi.nlm.nih.gov/geo/). The GSE61592 dataset included three normal heart samples and three MI/R injury samples. The GSE67308 dataset included four normal heart samples and four MI/R injury samples. The MI/R injury samples of two datasets were obtained from mice subjected to I/R involving left anterior descending coronary artery occlusion followed by reperfusion. The mice with MI/R injury had larger infarct size of left ventricle (LV), more serum cardiac troponin I, and lower LV ejection fraction (1,2). The raw data were first normalized and then, the Geoquery package was used to compare the gene expression between MI/R injury and normal samples.

### Identification of overlapping DEGs and enrichment analysis

After data preprocessing, the fold change (FC) of gene expression and the false discovery rate (FDR) was calculated between MI/R and normal samples. DEGs were identified with a |log FC| ≥2 and an FDR <0.05. Furthermore, the DEGs of GSE61592 and GSE67308 were compared to identify the overlapping DEGs. Gene ontology (GO) and Kyoto Encyclopedia of Genes and Genomes (KEGG) (3) pathway enrichment analysis were performed using the Database of Annotation, Visualization and Integration Discovery (DAVID; version 6.8; http://david.abcc.ncifcrf.gov/) (4). The GO terms and KEGG pathways with an FDR <0.05 were considered to be significantly enriched.

### Identification of PPI network

A PPI network of overlapping DEGs was established by the Search Tool for the Retrieval of Interacting Genes (STRING; version 11.0; http://string-db.org/) (5). The overlapping DEGs interaction pairs with a combination score >0.4 were analyzed for establishment of PPI network. Then, the integrated interaction information was obtained from STRING. The network was visualized and the score of each protein was calculated according to the evidence of protein interactions. Hub genes were identified with a combination score >3.

### Identification of potential miRNAs and small molecules

To identify potential miRNAs in MI/R injury, overlapping DEGs were analyzed in the microRNA.org database (http://www.microrna.org) (6), which is a comprehensive database of microRNAs target predictions and expression profiles. The database is based on the mirSVR algorithm, which trains regression models for sequences and context features extracted from target sites predicted by miRanda. The miRNAs with an FDR <0.05 were considered to be significantly related to MI/R injury.

To find potential therapeutic small molecules, overlapping DEGs were analyzed in the Comparative Toxicogenomics Database (CTD; http://ctdbase.org/) (7), which integrated interaction information between chemical and gene/protein by a hierarchical interaction-type vocabulary. The cutoff criterion was set as FDR<0.05.

## Results

### Identification of overlapping DEGs

**Figure 1:**
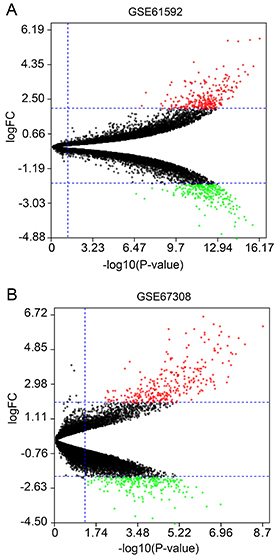
Volcano plot of differentially expressed genes in (**A**) GSE61592 and (**B**) GSE67308. The Y coordinate is log2 (fold change) and the X coordinate is -log 10 (P-value). Red dots represent significantly upregulated genes while green dots represent significantly downregulated genes. Black dots are genes of non-significant differences.

**Figure 2:**
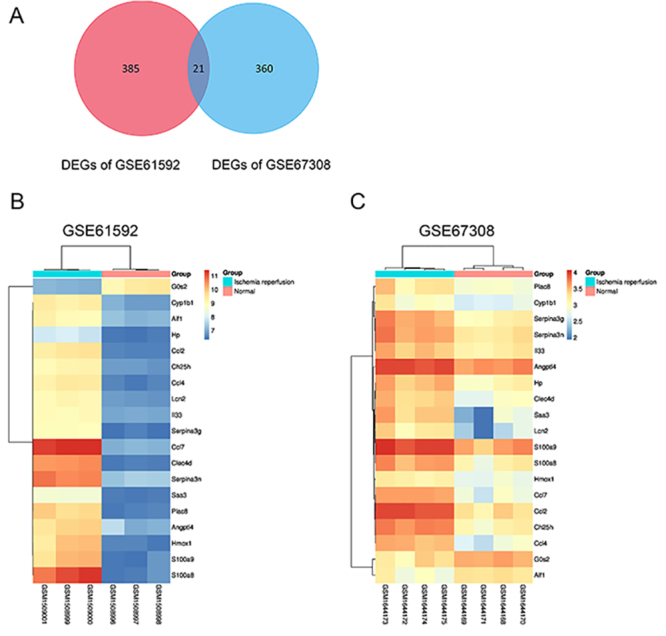
Venn diagram and hierarchical cluster analysis of overlapping differentially expressed genes (DEGs) in GSE61592 and GSE67308. **A**, The red circle represents DEGs of GSE61592 dataset and the blue circle represents DEGs of GSE67308 dataset. Overlapping differentially expressed genes are represented by the intersection of two circles. **B** and **C**, The cluster of the overlapping DEGs is displayed on the left of the heatmap while the cluster of the samples is displayed at the top of the heatmap. Gene symbols of overlapping DEGs are shown on the right of the heatmap. The accession numbers of samples from Gene Expression Omnibus database are shown at the bottom of the heatmap.

Based on the cutoff of |log FC| ≥2 and FDR <0.05, a total of 406 DEGs were identified between the MI/R injury and normal samples, including 219 upregulated genes and 187 downregulated genes in GSE61592 dataset. Three hundred and eighty-one DEGs were identified in GSE67308 dataset, including 224 upregulated genes and 157 downregulated genes. The identified DEGs are shown in a volcano plot (Figure 1). In the volcano plot, red dots represent significantly upregulated genes while green dots represent significantly downregulated genes. Then, 21 overlapping DEGs were identified after comparison of DEGs in two datasets. For the identified overlapping DEGs, hierarchical cluster analysis was performed. In Figure 2, the cluster of the DEGs is displayed on the left of the heatmap while the cluster of the samples is displayed on the top of the heatmap. The MI/R samples were obviously separated from normal samples, indicating the reliability of the overlapping DEGs.

### GO functional annotation and pathway enrichment analysis in overlapping DEGs

**Figure 3:**
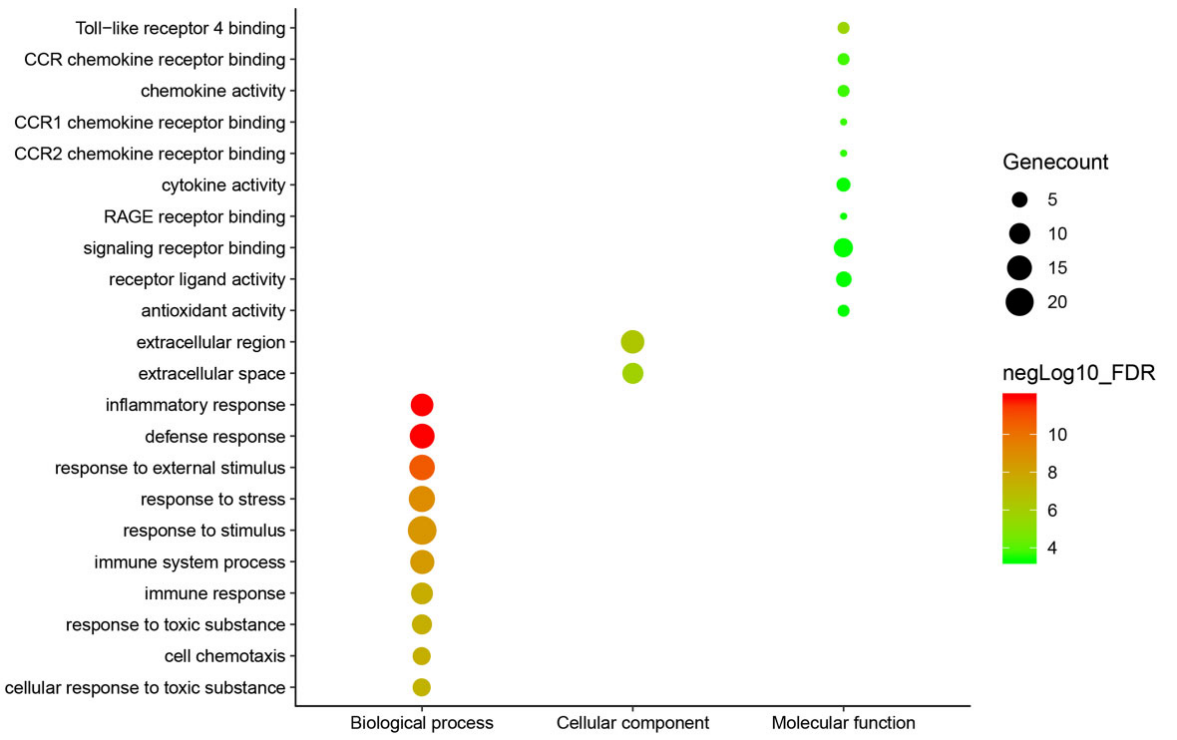
Gene ontology (GO) functional annotation enrichment analysis of overlapping differentially expressed genes. The X coordinate represents GO terms, including biological process, cellular component, and molecular function. The Y coordinate represents the names of GO terms. The red and green points represent high and low false discovery rate (FDR) values and the size of points indicates gene count.

The overlapping DEGs were analyzed by DAVID. These genes were significantly enriched in several GO terms related to biological processes, molecular function, and cellular components (Figure 3). The top 5 related biological processes were defense response (FDR: 1.13E-12), inflammatory response (FDR: 1.13E-12), response to external stimulus (FDR: 2.03E-11), response to stress (FDR: 1.09E-09), and response to stimulus (FDR: 2.44E-09). There was a significant correlation in the extracellular region (FDR: 4.15E-07) and extracellular space (FDR: 1.48E-06). In addition, the terms related to molecular function were mainly involved in toll-like receptor 4 binding (FDR: 2.41E-06), CCR chemokine receptor binding (FDR: 0.00019), chemokine activity (FDR: 0.00022), cytokine activity (FDR: 0.00041), and antioxidant activity (FDR: 0.00042). Multiple signaling pathways of co-DEGs were enriched, including interleukin (IL)-17 signaling pathway (FDR: 9.18E-07), chemokine signaling pathway (FDR: 0.0112), cytosolic DNA-sensing pathway (FDR: 0.0184), and cytokine-cytokine receptor interaction (FDR: 0.0184) (Table 1).

**Table 1:** Kyoto Encyclopedia of Genes and Genomes pathway enrichment analysis of overlapping DEGs.

Pathway	FDR	Gene count	Genes
IL-17 signaling pathway	9.18E-07	5	CCL2, CCL7, LCN2, S100A8, S100A9
Chemokine signaling pathway	0.0112	3	CCL2, CCL4, CCL7
Cytokine-cytokine receptor interaction	0.0184	3	CCL2, CCL4, CCL7
Cytosolic DNA-sensing pathway	0.0184	2	CCL4, IL33

DEGs: differentially expressed genes; FDR: false discovery rate.

### Construction of PPI network and identification of hub genes

**Figure 4:**
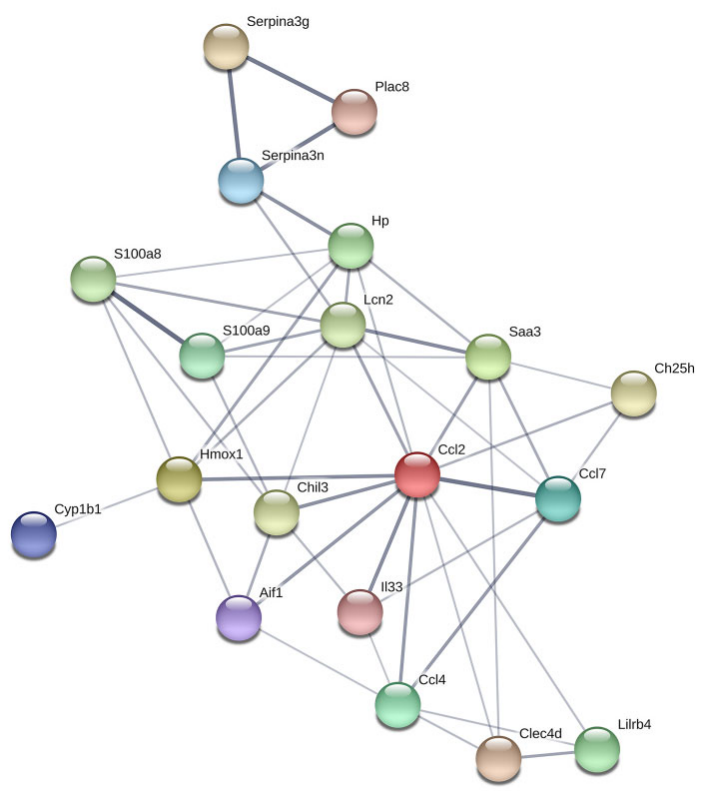
Overlapping differentially expressed genes in the protein-protein interaction network. The protein is represented by a node, and the interaction between paired proteins is represented by an undirected line.

Proteins related to overlapping DEGs were selected to establish the PPI network based on the STRING database (Figure 4). After calculating the score of each gene, 7 overlapping DEGs with a degree >3 were considered to be crucial for MI/R, including CCL2, LCN2, HP, CCL7, HMOX1, CCL4, and S100A8 (Table 2).

**Table 2:** Protein-protein interaction network degree of identified hub genes.

Genes	Degree	Up/down
CCL2	7.911	Up
LCN2	5.207	Up
HP	3.833	Up
CCL7	3.611	Up
HMOX1	3.419	Up
CCL4	3.120	Up
S100A8	3.018	Up

### Potential miRNAs and small molecules associated with MI/R injury

According to the MicroRNA.org database, the potential miRNAs of overlapping DEGs were screened out (Table 3). There were 6 identified potential miRNAs, including miR-24-3p, miR-26b-5p, miR-2861, miR-217, miR-4251, and miR-124-3p.

**Table 3:** Potential microRNAs of overlapping DEGs.

microRNA	FDR	Gene count	Target genes
miR-24-3p	1.93E-02	4	S100A8, CCL2, CCL4, HMOX1
miR-26b-5p	1.93E-02	5	CCL2, CCL7, PLAC8, HMOX1, CH25H
miR-2861	2.91E-02	3	AIF1, ANGPTL4, LCN2
miR-217	3.60E-02	4	CLEC4D, CYP1B1, CCL4, HMOX1
miR-4251	3.61E-02	4	CLEC4D, CCL7, PLAC8, LCN2
miR-124-3p	3.85E-02	4	CYP1B1, CCL2, ANGPTL4, HMOX1

DEGs: differentially expressed genes; FDR: false discovery rate.

miR-24-3p and miR-26b-5p were among the most significant miRNAs, and most miRNAs target CCL2 and HMOX1.

According to the CTD database, the DEGs were analyzed to find potential small molecule drugs. Several small molecules were screened out to have significant correlations with the overlapping DEGs (Table 4). The top 20 small molecules are listed in Table 4, such as pregnenolone carbonitrile, ozone, muraglitazar, doxorubicin, and troglitazone. Four small molecules were predicted to be potential drugs for MI/R injury, including ozone, troglitazone, rosiglitazone, and n-3 polyunsaturated fatty acids. CCL2, CCL4, S100A8, S100A9, LCN2, and HMOX1 can be targeted by several small molecules.

**Table 4:** Top 20 potential small molecules of the overlapping DEGs.

Molecule in CTD	FDR	Gene count	Target gene
Quartz	4.99E-10	7	S100A9, HP, CCL2, CCL7, HMOX1, LCN2, CH25H
Pregnenolone carbonitrile	4.99E-10	13	CLEC4D, S100A8, AIF1, S100A9, HP, CYP1B1, CCL2, IL33, G0S2, ANGPTL4, HMOX1, LCN2, CH25H
Ozone	2.40E-09	12	S100A8, S100A9, CYP1B1, CCL2, CCL4, IL33, CCL7, G0S2, ANGPTL4, HMOX1, LCN2, CH25H
Muraglitazar	5.64E-09	9	AIF1, CCL2, CCL4, IL33, CCL7, G0S2, ANGPTL4, LCN2, CH25H
Hexachlorobenzene	2.78E-08	7	S100A8, AIF1, S100A9, HP, CYP1B1, HMOX1, LCN2
Dimethylnitrosamine	4.03E-08	10	AIF1, S100A9, HP, CCL2, IL33, CCL7, PLAC8, G0S2, HMOX1, LCN2
Doxorubicin	5.77E-08	12	S100A8, AIF1, S100A9, CYP1B1, CCL2, CCL4, CCL7, PLAC8, G0S2, HMOX1, LCN2, CH25H
Troglitazone	7.00E-08	11	HP, CYP1B1, CCL2, CCL4, IL33, CCL7, G0S2, ANGPTL4, HMOX1, LCN2, CH25H
Naphthalene	8.36E-08	8	S100A8, S100A9, CYP1B1, CCL2, CCL7, ANGPTL4, HMOX1, CH25H
Isoproterenol	1.26E-07	10	CLEC4D, S100A8, AIF1, S100A9, CYP1B1, CCL2, CCL7, HMOX1, LCN2, CH25H
Cobalt	2.36E-07	7	CYP1B1, CCL2, CCL4, CCL7, G0S2, ANGPTL4, HMOX1
Rosiglitazone	2.92E-07	11	AIF1, HP, CYP1B1, CCL2, CCL4, CCL7, G0S2, ANGPTL4, HMOX1, LCN2, CH25H
Tesaglitazar	3.13E-07	8	AIF1, CCL2, CCL4, IL33, G0S2, ANGPTL4, HMOX1, CH25H
1-Naphthylisothiocyanate	6.76E-07	9	S100A8, AIF1, S100A9, CCL2, IL33, PLAC8, G0S2, HMOX1, LCN2
CI 1044	6.76E-07	5	S100A8, S100A9, CYP1B1, HMOX1, LCN2
Trimethyltin	6.76E-07	5	AIF1, CCL2, CCL4, HMOX1, LCN2
Chloroprene	1.22E-06	10	S100A8, AIF1, S100A9, CYP1B1, IL33, PLAC8, G0S2, ANGPTL4, HMOX1, CH25H
Trinitrobenzenesulfonic acid	1.69E-06	7	S100A8, S100A9, CCL2, CCL4, CCL7, HMOX1, LCN2
n-3 Polyunsaturated fatty acids	2.09E-06	5	HP, CYP1B1, CCL2, CCL7, HMOX1

DEGs: differentially expressed genes; CTD: Comparative Toxicogenomics Database; FDR: false discovery rate.

## Discussion

In this study, 21 overlapping DEGs were identified between normal and MI/R injury samples. Function annotation and pathway enrichment analysis of overlapping DEGs were then conducted. IL-17 signaling pathway, chemokine signaling pathway, cytosolic DNA-sensing, and cytokine-cytokine receptor interaction pathway were screened out according to the KEGG analysis. Furthermore, the PPI network of overlapping DEGs was constructed and CCL2, LCN2, HP, CCL7, HMOX1, CCL4, and S100A8 were selected as the hub genes based on the degree of connectivity. Potential miRNAs and small molecules associated with MI/R injury were predicted.

As shown in Table 1, the IL-17 signaling pathway involved the largest number of co-DEGs. Inflammation plays a crucial role in MI/R injury. As an important cytokine in cardiovascular diseases, the level of IL-17A increased after ligation and reperfusion of the left coronary artery in mice. IL-17A knockout or treatment with anti-IL-17A monoclonal antibody significantly attenuated I/R injury and improved cardiac function by reducing cardiomyocyte apoptosis and neutrophil infiltration (8). In contrast, treatment with exogenous IL-17A induced the opposite effect.

In the PPI network, upregulated CCL2, LCN2, HP, CCL7, HMOX1, CCL4, and S100A8 were identified to be hub nodes. Notably, CCL2, LCN2, CCL7, and S100A8 were enriched in the IL-17 signaling pathway, which suggested that these genes might play crucial roles in MI/R injury. Chemokine CC motif ligand-2 (CCL2), CCL7, and CCL4 are chemokines that are involved in inflammatory responses. CCL2 has been shown to be involved in the pathophysiology of ischemic heart disease; however, the specific effect of CCL2 on I/R injury remains controversial. CCL2 is induced in the endothelium of small veins immediately after reperfusion and its induction is confined to the previously ischemic area that has been reperfused (9). In the first 60 minutes after reperfusion, CCL2 and TGF-β1 promote infiltration of monocytes into formerly ischemic myocardium (10). However, CCL2 was also found to prevent LV dysfunction after global I/R through a reactive oxygen species-dependent but K (ATP) channel-independent pathway (11). The role of CCL2 might not be clear in MI/R injury, but it has effects on macrophage recruitment and activation, cytokine synthesis, and myofibroblast accumulation. There are few studies about the relationship between CCL7 and CCL4 and MI/R injury, but they might have effects on the inflammatory process and more research has to be done in the future.

Lipocalin-2 (LCN2) is upregulated in pathological conditions such as obesity, inflammation, hypertension, and cancer. Circulating levels of LCN2 are augmented and correlated closely with the severity of coronary heart disease. LCN2 could regulate the granulocyte infiltration and chemokine expression during MI/R. It is upregulated after MI/R injury, and anti-Lcn2 antibody treatment reduces neutrophil and macrophage infiltration, suppresses M1 polarization, and ameliorates MI/R injury (12). These findings collectively support a role of LCN2 in the pathogenesis of MI/R injury.

S100A8 and its dimeric partner S100A9 are pro-inflammatory molecules that are significantly increased one day after percutaneous coronary intervention in patients with acute MI, and elevated S100A8/A9 levels are associated with the incidence of major adverse cardiovascular events (13). In experimental research, S100A8/A9 has been identified as a master regulator causing cardiomyocyte death in the early stage of MI/R injury via suppression of mitochondrial function. Recombinant S100A8/A9 treatment increases myocardial injury and exacerbates heart failure in a mouse I/R model (14).

Although hub genes haptoglobin (HP) and heme oxygenase-1 (HMOX1) are not enriched in the IL-17 signaling pathway, they also play important roles in MI/R injury. HP is an abundant plasma protein that prevents oxidative damage and plays a role in immune regulation and reverse cholesterol transport. In MI, HP exerts important effects on both short- and long-term cardiac repair responses through reduction of oxidative stress, maintaining microvascular integrity, myocardial structure, and proper scar formation (15). Higher levels of plasma HP are independently related to poor overall survival in acute MI patients (16). As a stress response protein, HMOX1 might prevent cells from injury caused by oxidative and pathological stress via degradation of oxidant heme and production of antioxidant bilirubin and anti-inflammatory molecule carbon monoxide (17). The level of HMOX1 protein expression in patients with coronary heart disease (CHD) is reported to be significantly higher than that in patients without CHD (18). HMOX1 deficiency aggravates cardiac inflammation after ischemia in mice, while hHMOX1 gene therapy reduces inflammation after I/R in murine and porcine hearts (19). Moreover, overexpression of HMOX1 in cardiomyocytes prevents I/R injury via induction of autophagy, inhibition of apoptosis, and reduction of mitochondrial oxidation products (20).

miRNAs can regulate gene expression after transcription, and play key regulatory roles in MI/R injury. In the present research, potential miRNAs of co-DEGs were identified. In cardiomyocytes following I/R, expression of miR-24-3p is significantly increased and the level of miR-24-3p is negatively correlated with the ischemia marker HIF-1a. Overexpression of miR-24-3p could decrease cardiomyocytes apoptosis due to I/R injury through the Nrf2-Keap1 pathway (21). miR-26b-5p is associated with adverse cardiovascular outcomes in patients with ST-segment elevation MI (22). In mice with MI, miR-26b activates the MAPK pathway through inhibiting PTGS2, thereby reducing inflammation and improving myocardial remodeling (23). miR-2861 regulates necrosis in MI by targeting adenine nucleotide translocase 1. Knockdown of miR-2861 reduces H_2_O_2_-induced cardiomyocyte necrosis and protects the heart from I/R injury and necrotic cell death *in vivo* (24). Overexpression of miR-217 aggravates hypoxia-induced H9c2 cell injury via inhibiting silent information regulator 1 expression (25). However, the relationship between miR-4251 and MI/R has not been reported. In the pathogenesis of MI, miR-124 promotes MI/R-induced cell death and apoptosis in cardiomyocytes by targeting SphK1 (26). Notably, these miRNAs target co-DEGs that are involved in the IL-17 signaling pathway. Therefore, we speculated that miR-24-3p, miR-26b-5p, miR-2861, miR-217, and miR-124-3p might play important roles in MI/R injury.

Furthermore, the co-DEGs were analyzed in the CTD database, and several small molecules were predicted to correlate with MI/R injury. Ozone, which has been shown to act as an immune effector and a signaling molecule in physiological processes, has been used to treat many diseases. In the rat I/R injury model, ozone oxidative preconditioning was demonstrated to enhance antioxidant capacity and protect the myocardium from I/R injury through mitigating mitochondrial damage and cardiomyocyte apoptosis (27). Pretreatment with oxygen/ozone mixture might protect the heart from acute MI by locally increasing eNOS expression and subsequent endothelial progenitor cell recruitment (28). Moreover, oxygen/ozone might have antiarrhythmic effects against arrhythmias caused by MI/R (29).

Troglitazone and rosiglitazone belong to thiazolidinediones, which are synthetic PPAR-gamma agonists and act as insulin sensitizers to treat type 2 diabetes. However, PPAR-gamma has been considered to be a regulator of inflammation and ischemic responses in recent years. In nondiabetic pigs, chronic troglitazone treatment improves the recovery of LV systolic and diastolic function after regional I/R (30). However, acute treatment with troglitazone increases sensitivity to ventricular fibrillation in MI/R (31). Whether thiazolidinediones have pro-arrhythmic potential in clinical application needs further study. In rat models, rosiglitazone could reduce MI and improve systolic dysfunction induced by I/R injury (32), but the role of rosiglitazone still remains controversial in many clinical studies. The RECORD trial showed that rosiglitazone might increase the risk of heart failure but it did not increase the risk of overall cardiovascular morbidity or mortality in people with type 2 diabetes (33). In addition, a meta-analysis found that rosiglitazone is associated with increased risk of myocardial infarction and heart failure in patients with impaired glucose tolerance or type 2 diabetes, without a significantly increased risk of cardiovascular mortality. However, some clinical trials found no association between rosiglitazone and increased risk of heart failure and myocardial infarction. Furthermore, rosiglitazone might reduce nitrosative stress, inflammation, and risk of the primary cardiovascular composite outcome and cardiovascular death (34).

The n-3 polyunsaturated fatty acids (PUFAs), which are mainly found in marine animals and plants, have immunomodulatory, anti-inflammatory, anti-arrhythmic, and anti-thrombotic properties. In the last few years, many animal studies have shown that n-3 PUFAs might have good effects on MI/R injury. In a rat model of acute MI/R, intravenous injection of n-3 PUFAs before reperfusion reduced vascular failure and shock caused by I/R (35). A diet rich in n-3 PUFA from fish oil affected heart function and improved cardiac responses to I/R via reducing oxygen consumption and increasing post-ischemic recovery (36). However, in clinical studies, there is still a lot of controversy about the role of n-3 PUFAs in cardiovascular diseases. Some trials found that n-3 PUFAs could not reduce the rates of serious coronary events and of coronary revascularization, and might even increase the risk of cardiovascular disease and diabetes in overweight adults (37). However, a large clinical trial showed that n-3 PUFA could significantly decrease the risk of death and cardiovascular death in 11,324 patients surviving recent (≤3 months) myocardial infarction (38). Other trials indicate that n-3 PUFAs might protect against atrial fibrillation, enhance stability of atherosclerotic plaques, reduce heart rate and blood pressure, and improve cardiovascular risk factors (39,40).

In conclusion, our study identified overlapping DEGs between GSE61592 and GSE67308, and these DEGs were enriched in significant pathways such as IL-17 signaling pathway. Identified hub genes like CCL2, LCN2, HP, CCL7, HMOX1, CCL4, and S100A8 and potential miRNAs (miR-24-3p, miR-26b-5p, miR-2861, miR-217, and miR-124-3p) might play vital roles in MI/R injury. Furthermore, four small molecules (ozone, troglitazone, rosiglitazone, and n-3 PUFAs), which might have positive effects on MI/R injury, were screened out. Considering the controversies in clinical trials, these small molecules as therapeutic drugs must be studied more in the setting of MI/R injury and other cardiovascular diseases. However, there were some limitations to the present study. These results were obtained only through bioinformatics analysis, and they were not demonstrated by real-time polymerase chain reaction or animal models. Although the overlapping DEGs, hub genes, potential miRNAs, and drugs were identified for MI/R injury, a study with bioinformatics analysis is just the first step and there is still a long way to translate these findings into clinical application. Despite these limitations, the results might provide new insights into the molecular mechanism, therapeutic targets, and potential drugs of MI/R injury.
